# Supplementary material for: Are carbon emissions trading and green financial instruments synergistic? -Comprehensive quantitative research based on content analysis
Source: PLoS One. 2024 Mar 7;19(3):e0298601. doi: 10.1371/journal.pone.0298601 (PMC10919601; doi:10.1371/journal.pone.0298601)
Supplement: S1 Data — (ZIP) [file pone.0298601.s001.zip › Data/地级市Z_F工作报告.zip!/╜¡╦╒/╤∩╓▌╩╨/└┤╫╘╣1⁄2╓┌║┼í╛┬φ┐╦╩2╛▌═°í┐_2003.docx]

各位代表：

现在，我代表扬州市人民政府，向大会作政府工作报告，请予审议，并请市政协各位委员和其他列席人员提出意见。

过去五年和2002年工作的回顾

五年来，我们在中共扬州市委的领导下，坚持以经济建设为中心，进一步深化改革、扩大开放，加快经济结构调整，加强城市建设和管理，促进了经济持续增长、社会全面进步、人民生活水平稳步提高，为向第三步战略目标迈进奠定了坚实的基础。

过去的五年，全市经济综合实力显著增强。五年中，国内生产总值上了两个台阶，1998年超过400亿元，2001年超过500亿元，2002年达到558.93亿元，与1997年相比，国内生产总值增加182亿元，增长60.6％；人均国内生产总值1.23万元，增长58.1％。五年累计完成固定资产投资695.1亿元，比前五年增长48.1％；累计实现财政收入185.34亿元，增长100.9％。

过去的五年，全市改革开放取得新的进展。稳步推进了国有企业、商贸流通、医药卫生、财税、住房、农村税费、政府机构、行政审批制度等各项改革，所有制结构有所调整，经济活力明显增强。亚星客车、扬农化工股票上市。外向型经济加速发展。五年累计外商直接投资5.3亿美元，比前五年增长27.7%;实际出口29.3亿美元，增长76.9％；外经营业额2.6亿美元，增长310%。非公有制经济迅速崛起，实现增加值已占全市经济总量的64.9%。

过去的五年，全市城乡面貌出现重大变化。五年共完成基础设施投入345亿元，比前五年增长46.8％。南绕城高速公路、京沪高速扬州段、宁通高速扬州段改造等相继竣工通车，实现了“县县通高速”；润扬长江公路大桥、西北绕城高速公路、宁启铁路扬州段、南水北调等工程相继开工。扬州二电厂一期工程建成投产，城网、农网改造基本完成。建成了全市城域宽带网。完成了江堤达标和引江河扬州段等重大水利设施。各县（市）城区和中心镇规模扩大，基础设施和配套功能继续改善。耕地资源得到有效保护。四县（市）先后成为全国生态示范区。

过去的五年，全市各项社会事业全面进步。累计实施省级以上“火炬”、“星火”项目249个，其中国家级129个。现有省级以上高新技术企业86家，其中国家级15家。大力推进素质教育，教育教学质量全面提高；累计改、扩建校舍35.3万平方米，基本实现了农村中小学无危房的目标。新建扬州大剧院、档案馆，完成体育场改造等工程。创作了一批群众喜闻乐见的文艺和广播电视精品，广陵书社批准建立，《扬州文化丛书》获中国第13届图书奖，有2个（件）作品获省以上“五个一工程奖”，新增省以上文保单位15个（处）。人口与计划生育、农村和社区卫生工作得到加强。

过去的五年，全市人民生活水平明显提高。与1997年相比，2002年城镇居民人均可支配收入增加1941元，农民人均纯收入增加794元，年均分别增长6.6％和4.6％；城乡居民储蓄余额增加194亿元，年均增长15.5％。城市居民人均住房使用面积达16.9平方米，增加7.1平方米；农民人均住房面积37.9平方米，增加10平方米。电话普及率达每百人26.8线。调整行政区划，改革户籍管理制度，城市化率预计达45％。社会保障体系初步建立，48.06万人参加养老保险。社会治安持续稳定，人民生活总体上达到小康水平。

2002年是我市经济和社会发展较快的一年，是“九五”以来各项指标完成得最好的一年，也是城市面貌变化最大的一年。我们紧紧围绕市第四次党代会和市四届人大五次会议确定的目标任务，举全市之力招商引资，加快改革和调整的步伐，加速改善投资环境，经济建设和社会事业呈现出良好的发展态势。全年国内生产总值558.93亿元，按可比价计算增长11.1%；财政总收入55.61亿元，增长36.6%，其中地方财政收入30.04亿元，增长55%；城镇居民人均可支配收入7833元，农民人均纯收入3920元，分别增长12.6%和6.2%；年末城镇登记失业率4%；人口出生率7‰。

招商引资再创新高，投资环境明显改善。〖HTF〗应对中国加入WTO，大规模、全方位走出去推介扬州，举办“烟花三月”经贸旅游节，承办江苏农业国际项目合作洽谈会，投资环境改善取得明显实效。全年直接利用外资2.56亿美元，同口径增长2倍多；合同利用外资5.1亿美元，增长1.7倍。实际出口8.01亿美元，增长20.6％。实现外经营业额7500万美元，增长16%。全年引进民资45亿元。完成全社会固定资产投资180.22亿元，增长25.1%。投资结构不断优化，私营个体经济投资占全社会投资总额的25％;生产性投入占45.3％。沿江开发和市、县（市）开发区、园区开发面积扩大，基础设施投入超过20亿元，一批有技术含量、投资规模的外资、民资项目在园区落户；以市经济开发区为龙头、五园和各县（市）开发区为重点、乡镇工业园区为支撑的开发开放新格局正在形成。西北绕城高速公路、润扬长江公路大桥及其北接线、宁启铁路扬州段共完成投资13.08亿元；沿江高等级公路和扬州港3万吨级件杂货码头已开工建设。一批国债项目按时竣工。

结构调整力度加大，发展活力得到增强。积极推进工业企业改制，国有股比重由年初的63%降至53%；扬农化工股票成功上市，联环药业股票上市申请已获通过。加大工业资源整合力度，市直调整设立了机电、化工、纺工3个资产经营管理公司；上汽仪征公司、江都宏运车业等一批资产重组项目运作良好，江扬船厂等一批难题得到有效破解。工业技改投入65亿元，增长26%。全部工业产值超千亿元，达1004.7亿元;规模以上工业实现增加值180亿元，增长11.2％。完成建筑业增加值35.2亿元，增长22.6%。农业和农村经济结构进一步优化。调减粮食种植面积30万亩，蔬菜、花木等和以特禽特畜特水为重点的种养业发展加快，建成一批标准化示范区和无公害农产品生产基地。农业利用外资981万美元，民资、工商资本2.35亿元。乡镇工业增加值、利税等均增长15%以上。农村外出劳务人员新增5.79万人，累计输出55.59万人。巩固了农村税费改革成果，化解村级债务6.5亿元，农民卡内负担下降3.6%。培训了200个村的村干部，增强了带领群众致富奔小康的能力。土地经营取得重大突破。农业资源开发和水利建设得到加强，气象和农机服务水平进一步提高。服务业发展势头良好。全年实现三产增加值215.5亿元，增长11%；实现社会消费品零售总额182.76亿元，增长10.3%。房地产业、信息服务业增长较快。旅游业持续发展，预计全年接待境外游客12万人次、国内旅游者630万人次，实现总收入52亿元，分别增长19％、14.1％和18％。

“三创”齐抓、“三金”齐夺，城乡面貌大为改观。以创建“国家卫生城市、国家环保模范城市、全国文明城市工作先进城市”为抓手，推动名城建设和投资环境、人居环境的改善。进一步调整城市总体规划。市区完成城建投入56亿元，其中城市基础设施投入13亿元，新改建道路68公里，拆迁房屋80多万平方米，新增绿化面积150万平方米,翻建街巷68条。启动西部新区建设，建成国际展览中心及部分配套设施。老干部活动中心封顶，体育中心二期改造、扬州中学综合大楼等一批工程完工。四水厂二期10万吨工程、汤汪污水处理厂一期10万吨工程、瘦西湖引水工程竣工投运，疏浚了小秦淮河等9条河流，城市水环境质量进一步好转。赵庄垃圾处理场建成并交付使用。中德合作扬州生态规划与管理项目进展顺利。结合区划调整，初步形成了“两级政府、三级管理、四级网络”的城市管理新体制。经过全市人民的共同努力，“三创”取得了一年夺“三金”的佳绩。我市还被评为全省社会治安综合治理先进城市，市区和江都、仪征分别获得省级文明城市称号。

各项社会事业全面发展，关注民生成效显著。九年义务教育得到巩固和提高，初中毕业生升学率88.9%，比上年提高9个百分点。完成了扬州职大与电大、教育学院与扬州师范、高邮师范的合并。改革卫生监督和疾病预防控制体制，在部分医院进行了经营权与所有权分离等改革试点。农村合作医疗覆盖率达40%，新增农村自来水受益人口8.47万人、改厕6.84万户。计划生育率99.3%，计生服务水平得到提高。完成市区广播电视资源的整合，提高了农村广播电视覆盖率。成功举办了市首届农民艺术节、第四届少儿艺术节、扬剧节和扬州中学百年校庆、纪念史可法诞辰400周年、“扬州八怪”国际研讨会，以及宝应荷藕节、高邮双黄鸭蛋节、江都国际龙狮邀请赛等活动。在省十五届运动会、六届残运会上，实现了金牌、总分超上届的目标；承办了第十八届全球华人篮球邀请赛、全国艺术体操冠军赛等重大赛事。各级政府贯彻落实市1号文件精神，围绕基本生活、基本养老、基本医疗、基本住房和再就业、贫困子女就学，加大了关注民生工作力度。巩固“两个确保”成果，企业离退休人员基本养老金社会化发放率达100%；城市居民低保对象基本做到应保尽保。就业和再就业工作取得新进展，安排就业、再就业岗位4.6万个。稳步推进医疗保险制度改革，城镇医保覆盖率达69%。一部分困难群众的住房问题有所缓解。帮扶农村186个经济薄弱村、1万多户贫困户初见成效。

各位代表，回顾过去的工作，我们在组织实施市委决定、市人大决议和履行政府职责的过程中，有以下几点做法和体会：

一是坚持以经济建设为中心，把发展作为第一要务。千难万难，发展经济就不难；千条线万条线，发展是主线。面对激烈竞争的态势和经济工作中的矛盾与问题，我们以“三个代表”重要思想为指导，以“富民强市”统揽全局，始终扭住经济发展不放松，实行“三资”联动、“三改”齐抓、“三创”并举，狠抓促进发展的各项措施落实，一些难点问题得到初步化解，经济呈现出良好势头。同时也进一步转变了人们的观念，振奋了人们的精神，一心一意抓经济、凝心聚力谋发展的氛围正逐步形成。

二是坚持突出重点抓关键，推动全局工作顺利开展。抓住重点就把握了全局，掌握了工作的主动权。我们在经济工作中，以招商引资为抓手，以园区建设为载体，以沿江开发为突破口，以改进服务为重点，着力打造“飞机”降落的跑道，引进了一大批外资、民资项目；在优化环境中，以“三创”为抓手，重点突出城市基础设施建设、环境综合整治和市容管理，实现了一年夺“三金”的目标；同时正确处理改革、发展、稳定的关系，加强社会治安综合治理，营造安定的社会环境，树立了城市新形象，进一步增强了广大群众和投资者的信心。

三是坚持以民声为第一信号，努力为群众谋利益办实事。实践“三个代表”重要思想，必须把执政为民、为民谋利作为工作的出发点和落脚点。我们通过设立市长信箱、办好扬州热线“寄语市长”栏目、征集群众建议、处理来信来访和深入基层察访等措施，倾听民意，了解民情，拓宽了与群众联系的渠道；对群众反映的热点难点问题，协调各方力量，积极加以解决，以纾解民困；对为民办实事项目，加强督促检查，不打“白条”，把实事办实、好事办好，更好地凝聚了民心和力量。

四是坚持不断开拓创新，努力形成务实高效的良好政风。我们坚持与时俱进、务实创新，弘扬团队精神,保持了工作上的高压态势和高效运转，提高政府工作的组织程度；坚持转变政府职能，推进经营城市、产业运作，优化投资环境，提升行政效率；坚持依法行政，接受人大法律监督和工作监督、政协民主监督以及广泛的社会监督；坚持秉承“亲商、安商、富商”和“亲民、安民、富民"的理念，改进作风，改善服务，形成了“说了算、定了干、一抓到底、抓出成效”的风气，形成了发展的氛围、干事的氛围、团结的氛围。

各位代表，过去五年我市经济建设和社会发展所取得的成绩，是全市人民在中共扬州市委的正确领导下，坚决贯彻党的基本理论、基本路线、基本纲领，同心同德，共同拼搏的结果。在此，我谨代表扬州市人民政府，向全市人民、向关心和支持扬州发展的同志们、朋友们、投资者们及各界人士致以崇高的敬意和衷心的感谢！

在充分肯定成绩的同时，我们也清醒地看到工作中存在的问题和不足。主要是：经济持续增长的后劲不足，企业改革改制不彻底，重点企业支撑力不强；投资结构不尽合理，生产性投入比例还不够高；招商引资地区间不平衡性较大，投资大、关联度高的项目不多；农业结构调整力度不大，农民增收难度较大；城市及园区基础设施还不完善，对经济和社会发展的承载力不强；城市长效管理水平还不高；民生方面还有一些问题亟待解决。我们要倍加珍惜已经形成的局面，发扬成绩，正视困难，克服不足，齐心协力推动扬州新发展。

今后五年发展目标

今后五年，是全面建设小康社会、推进社会主义现代化进程的关键五年，是建设“经济强市、文化大市、旅游名市、生态园林城市”、实现新跨越的关键五年，是实现富民强市、在苏中率先崛起的关键五年。我们必须抓住二十一世纪头20年大发展、大开放、大开发的重要战略机遇期，大力实施外引内联、沿江开发、城市化、科教兴市、可持续发展五大战略，把扬州建设成为古代文化与现代文明交相辉映的名城。为此，必须抢抓四个难得的机遇：

一是抢抓国际资本和产业向长江三角洲集聚的机遇。扬州处在京杭大运河与长江、京沪高速公路与宁通高速公路、沿沪宁线IT产业带与沿江化工产业带的交汇处，随着润扬长江大桥等基础设施的建成，区位优势将进一步凸现，一些化工、汽车以及传统产业的升级项目正向扬州转移，为资金、产业、技术、人才的集聚提供了平台，带来了契机和活力。我们应该努力打造载体，提升对资本、产业等承载能力，使我市成为“长三角”继浦东、苏南以后的又一个投资热点地区和产业集群地区。

二是抢抓全面建设小康社会、加快推进社会主义现代化的机遇。十六大提出的新时期全面建设小康社会的目标，孕育着一种新的机遇，引发经济发展的新活力，必将掀起新一轮的思想解放和发展高潮。我市人均国内生产总值已达1490美元，正处于经济结构、产业结构、消费结构的大变动时期，应该乘势而上，构筑新的发展平台，力争在5年内基本达到全面小康水平。

三是抢抓区域经济一体化的机遇。以上海为龙头的“长三角”15个中心城市，正在谋求扩大合作、错位竞争、共同提升的发展路子，加速经济一体化。北京“办奥”、上海“办博”，形成了我国经济发展的南北两大助推器，必将强劲拉动“长三角”地区经济和社会的发展。我市处在上海经济圈、南京都市圈之中，在区域合作和资源整合中，完全可以谋取更大的发展空间，赢得更多的发展机会。

四是抢抓沿江开发的机遇。省委、省政府已把加快苏中沿江开发作为新世纪、新阶段江苏提升苏南水平、促进苏中崛起、带动苏北发展，实现大跨越的重要战略举措。我市有长江岸线80多公里，沿江可供开发的腹地大，这些宝贵资源也是率先崛起的潜在优势。我们应该加快沿江开发的步伐，加快资源整合，变资源优势为现实的经济优势，增强综合竞争力和经济实力。

今后五年调控的指标初步安排为：

国内生产总值年均增长12%，到2007年达到1000亿元，其中，一产、二产、三产年均分别增长6%、13.5%和13%；财政收入年均增长12-15%，期末超过100亿元；固定资产投资年均增长15%，累计完成投资超1000亿元；社会消费品零售总额年均增长11%；城镇居民人均可支配收入和农民人均纯收入分别年均增长8%和6%。

今后五年重点在以下几方面求得新发展：

1、经济总量、经济素质明显提高。到2007年，人均国内生产总值达到2.2万元（折合2650美元），继续保持苏中领先地位；全市财政总收入占国内生产总值的比重达到10%以上；三次产业结构更加优化。

2、经济国际化、信息化、工业化程度明显提高。直接利用外资年均增长30%，累计利用外资30亿美元；年均利用民资60亿元，累计超300亿元；沿江地区和园区直接利用外资占全市80%，出口年均增长30%以上。培育一批技术含量高、市场容量大、投资规模大的企业和新兴产业，形成经济新的增长极、产业新高地。

3、城市化程度明显提高。城市基础设施和功能设施完善，环境优美、生态良好。到2007年，市区绿化覆盖率达40%，污水处理率达80%；全市城市化率达49%以上，成为长江三角洲地区最适宜创业的城市、最佳人居城市之一。

4、城乡居民生活水平和质量明显提高。到2007年，人民生活更加富裕，城镇居民、农民住房大为改善，建成一批现代化的教育、文化、卫生、体育设施。城镇登记失业率控制在4.5%以内。

5、市场经济体制基本建成。基本形成适应社会主义市场经济的管理体制、运行机制；基本形成较为完善的资本、劳动力、土地等生产要素的市场体系；基本形成覆盖全社会的社会保障体系。

2003年政府工作任务

今年政府工作的总体要求是：以邓小平理论和“三个代表”重要思想为指导，认真贯彻落实党的十六大精神，围绕“富民强市、率先崛起，全面建设小康社会，推进社会主义现代化建设”的目标，坚持发展第一要务不动摇，加大改革开放和结构调整的力度，加快沿江开发和园区建设，加速资源集聚和有效投入，积极建设“文化扬州”、“生态扬州”、“实力扬州”，提高经济持续发展能力，提高城市综合竞争能力，提高人民群众物质文化生活水平，实现全市经济和社会事业的新突破、新发展。

国民经济和社会发展目标初步安排是：国内生产总值增长12%；财政收入增长15%以上；固定资产投资增长16%以上；直接利用外资增长60％以上；引进民资增长40%以上；实际出口增长15％；城镇居民人均收入增长8%，农民人均收入增长6%，城镇登记失业率控制在4.5%以内；人口出生率控制在10‰以内。完成上述目标任务，突出抓好以下几方面工作：

1、以沿江开发、园区建设为突破口，全面推进开发开放新跨越。我市今后经济发展的潜力在沿江，重点在园区。沿江开发是大思路、大决策、大战略，我们要进一步形成共识，强力推进，打造沿江的经济带、产业群，推动扬州经济从“运河时代”走向“长江时代”，扬州城市从“江北城市”走向“滨江城市”。今年定为“沿江开发年、园区建设年、项目投入年”。

（1）加快编制沿江开发规划，科学确定产业布局。抓紧编制沿江规划建设区和控制区范围内的沿江开发总体概念规划；编制沿江岸线利用、港口开发、土地利用、产业布局、交通设施、生态环境和发展组团中心镇等分项规划。建立和完善市、县（市）区沿江开发协调领导机构，按照“统一规划、分级开发”的要求，组织有序开发。按照“区域集中、能量集聚、产业集合、开发集约”的要求，加快打造沿江六大产业板块和三个群体：一是纺织板块。以市开发区纺织工业园和纺织出口加工区为载体，以仪征化纤为龙头,以高档纺织品为目标，形成上下游垂直整合的纺、织、染、后整理及服装加工的产业链。二是化工板块。在市开发区沿江发展精细化工和生物、医药、日用化工产业；在仪征沿江发展重化工、石油化工和新型化工材料等产业。三是汽车及船舶板块。汽车制造以亚星集团、上汽仪征公司、江都宏运汽车为依托，建成国内大中型客车、专用车和汽车零部件生产基地。船舶制造以大集团为依托，整合中小造、拆船企业，形成船舶制造基地。四是高科技产业板块。重点进行信息电子、生物工程、新材料、机电一体化等高科技研发和生产制造。五是生态农业和林业板块。建设沿江生态农业、水产业园区，形成种植养殖产业群、农产品深加工基地和城市森林、生态网络。六是物流旅游服务业板块。统一打“扬州港”牌子，形成以扬州港为依托、以仪征、江都港为作业区的“一港二区”格局，建设集水路、公路、铁路于一体，以集装箱、木材杂货、化工为重点的区域性多功能物流中心。规划建设古运河景观线、长江风情旅游线和一批景区景点。通过规划引导和高起点开发、有序开发、可持续开发，在沿江地区逐步形成港口群、产业群、城镇群。

（2）加速推进沿江开发和园区建设，完善基础设施配套。加大园区建设力度。在沿江地区规划好一批产业特色明显的园区，形成适宜大项目降落的“跑道”。省级开发区抓好扩容、升级工作，全力加快出口加工区报批。加快水、电、气、路和通讯网络等基础设施建设。完成沿江高等级公路东至京杭大运河、西至仪征胥浦河段的建设。改造扬子江路、扬瓜路。规划改建扬圩路、江都通港路、仪征万年大道等，形成与沿江高等级公路相衔接的“一横多纵”的路网体系。市开发区开工建设热电联供工程、污水处理厂、海关直通点。建成邗江工业园、港口和火车站的变电所。加快长江岸线整治。加大各县（市）开发区和乡镇工业园投入，完善基础设施和服务功能。积极推进项目建设。严格按照产业规划，优化生产力布局。对在手的元太科技、英群电子、华夏光电、华芮服装、大连化学等1000万美元以上的项目，提供优质服务，确保项目早建设、早投产。主攻一批产业关联度大、投资规模大、科技含量高项目以及能大量安排就业的劳动密集型项目。全市开发区和各类园区年内新批1000万美元以上项目30个。

（3）强化招商攻势，实现引资新突破。继续举全市之力招商引资，确保直接利用外资4亿美元，民资60亿元。一是在区域上主攻台湾、韩国，拓展日本、东南亚，兼顾欧美。组织系列推介招商活动，积极拓展信息源、项目源，力争在一些重大项目上取得新的突破。二是重点组织以沿江开发为专题的招商攻势，大密度、高强度推介沿江和工业园区。通过组团式招商、委托招商、中介招商、驻地招商、网上招商和以外引外等方式，瞄准国内外大集团、大企业或上市公司，加强同仪化、江苏油田、扬子石化等企业的产业合作招商。沿江地区直接利用外资占全市的70%左右。三是精心组织中国·扬州“烟花三月”国际旅游节暨经贸月活动。办好扬州产品展、投资环境展，做大扬州城市品牌，吸引更多的客商来扬投资创业。四是继续改善投资软环境。着力解决外来投资中商务、事务上存在的困难，继续推行服务承诺制。加大督查、考核和项目跟踪力度，落实领导责任制，确保项目落地。

2、以扩大投资、培植后劲为重点，加快构筑经济发展新平台。

扩大投资仍是拉动经济增长的有效途径。必须以资产重组、资源整合、资本经营、资金盘活为手段，加大投资、整合力度，集聚能量，加速经济的裂变和聚变。保持对重点领域和重点项目的强势投入。突出沿江开发，突出交通、能源、水利、农业等重大基础设施建设，突出城市建设，突出工业项目投入，突出商贸旅游项目建设。抓好润扬大桥、西北绕城公路、宁启铁路扬州段及火车站、南水北调东线一期、西气东输、沿江高等级公路等工程，以及县城功能性基础设施工程建设。全社会固定资产投资总额达到210亿元。努力扩大外资、民资在全社会投资中的比重。进一步拓宽外资、民资进入基础设施、教育、卫生、旅游等领域的渠道。鼓励有实力的非政府投资主体参与城市和小城镇建设、园区建设，吸引境内外大企业、大集团和国内有实力的上市公司与我市企业实行资产重组。继续大规模引进外地民资，发动千家万户兴办实业，鼓励在外创业的企业家“钱回流、凤还巢”；大力推进品牌化战略，培植外向型、科技型民营企业，推动民营经济上规模、上水平。积极争取国家和省各种政策性投入。抓住国家投资政策向重大项目倾斜、向改善农村生产条件倾斜、向结构调整倾斜、向生态环境建设倾斜的机遇，包装、组合一批重点项目，积极向部、省申报，争取二电厂二期工程等一批项目列入国家计划。加快投融资体制改革。加大财政资金筹集、支持力度，扩大融资性投入。改革投融资体制，整合政府有效资源，组建市投资发展公司，盘活存量资产，增强投融资能力；加快国有资产经营公司的机制改革，实行企业化、实体化运作；对新建的重大项目，建立项目公司或项目法人制，独立运作。对土地实行集中统一管理，依法搞活土地经营。对国家和省要求配套贴息的项目，落实配套政策。加强银企沟通与协作，鼓励各商业银行加大对重点项目和中小企业的信贷支持，创建金融安全区。

3、以优化结构、改组改制为主线，推动经济提速上新水平。

（1）实施“双改双增工程”，加快工业经济发展。工业是全市经济的主要支柱，没有工业的加快发展和质态改善，就难以支撑全市经济的持续快速增长。必须坚持走新型工业化的道路，实施“改革调整增活力、改造提高增后劲”的“双改双增工程”，在企业改革改制、项目投入、园区开发、重组整合上求突破，确保完成工业经济增长目标。加大技改投入，推进信息化带动工业化。狠抓200个投资千万元以上的重点技改项目实施进度，确保仪化45万吨PTA、琼花集团智能卡基材等一批项目竣工投产，扬农氟啶脲、英谛公司车用空调等一批项目开工建设。加快推进产品创新，重点抓好金方圆数控柔性加工单元、亚星新型城市客车等100项新产品的开发、投产。优先发展信息产业，实施20项信息化带动工业化技术创新项目。积极推进企业上网和电子商务工作，培植20户重点信息化示范企业。鼓励企业运用高新技术和先进适用技术改造传统产业，年内培育5个省以上高新技术企业和50个省以上高新技术产品。加快结构调整和资源整合。采取做大做强、引大引强、靠大靠强等多种措施，培育重点骨干企业。加速推进市直工业一批资产重组项目。完成联环药业等企业的股票上市工作，培育一批上市后备企业。积极鼓励市直工业企业以多种形式参与纺织工业园、化学工业园、汽车产业园等园区的建设。狠抓企业改革改制攻坚。加快市直50家困难企业的改制进度，年底完成80%左右。多渠道筹措改革成本，加快职工劳动关系和产权“双置换”。改制企业除少数经批准的外，国有股一律不再控股，国有股比重再降15个百分点左右。进一步推进现代企业制度建设，完善法人治理结构和激励约束机制，加强企业管理，降低成本，提高效益。完善国有资产经营管理体制，放手让资产经营公司大胆经营管理，确保国有资产保值增值。充分发挥江苏华建股份公司的龙头作用，推动建筑业扩大规模上水平。

（2）加快农业结构调整，促进农村经济全面发展。全面建设小康社会，必须更多的关注农村，关心农民，支持农业。以致富农民为目标，实施为期3-4年的“农民人均增收千元、全面奔小康计划”，今年在农业结构调整和产业化经营、乡镇工业园区、小城镇建设上迈出新的步伐。加快农业区域布局和农业结构调整，推动农业向产业化、区域化、市场化、标准化方向发展。到年底累计建成无公害农产品基地80万亩、花木林地面积80万亩、蔬菜播种面积100万亩、水产养殖面积100万亩。充分发挥中国农科院家禽研究所、扬州农科院和扬大农学院的优势，加快推广适用科技成果和新品种，提高农产品质量安全水平。新增无公害农产品和绿色食品品牌50个以上。加快建设现代农业产业园，推动“三资”农业发展。包装农业招商项目，扩大在境内外招商，抓好“农洽会”项目的跟踪落实，全年农业利用外资2000万美元，利用民资和工商资本4亿元。抓好各具特色的现代农业产业园和农业产业化示范工程，新建市现代科技农业示范园，提高农技、农机、信息服务水平；建设区域特色农产品市场，积极发展农民专业合作经济组织和农民经纪人队伍；组织农业名特优新产品博览会，进一步扩大沪扬、宁扬农业经济合作；发展专业村、专业户，抓好特色农产品加工和出口。加强农业水利建设，加强耕地保护和复垦工作，提高农业可持续发展能力。突出抓好乡镇工业园区，推动小城镇建设。大力吸引各类资本投入乡镇工业园区，推进集群发展。加强乡镇企业技术创新和机制创新能力，增强其发展后劲。进一步调整完善小城镇和农村片区建设规划。建立小城镇建设的多元投入机制，有序引导农民进镇，经过几年的努力，把全市的重点镇和中心镇建设成为经济繁荣、布局合理、功能健全、环境优美、富有地方特色的新型小城镇。开辟多种渠道，吸纳和输出劳力10万人以上。

（3）发展提升服务业，大力繁荣城乡经济。以建设区域性的商贸城市和旅游名市为目标，强化规划整合、强化业态创新、强化品牌意识，加大改制改组、招商引资、宣传促销力度，整顿市场秩序，扩大规模，争创优势。加快发展商贸流通业。大力发展新型流通业态，有计划引进国内外大型连锁企业；鼓励知名商贸企业发展连锁经营、规模经营、品牌经营。提高文昌阁中心商贸圈建设水平；规划启动西部商贸中心。改造和提升传统饮服业，积极申办中国淮扬菜烹饪大赛，扩大淮扬菜的影响和辐射。加强市场建设，提升档次，扩大规模，力争实现市场成交额300亿元，增长12%以上。加快商贸流通业改革，年内基本完成市直流通企业改制任务。调整优化出口产品和市场结构，进一步扩大外贸出口。做大做强旅游业。以文化、生态为特色，积极完善旅游总体规划和重大项目的规划，抓好一批市、县（市）区重点旅游项目开发。打造好“烟花三月”国际旅游节品牌，办好中国江都民间歌舞大赛、第二届邮文化节、宝应荷藕节等；利用鉴真东渡1250周年纪念活动，推出宗教文化游。整合旅游资源，成立市旅游业资产经营公司或旅游集团，实行政企分开。全年接待境外游客14万人次，国内旅游者730万人次，均增长15%以上。发展新兴服务业。加快以宽带网为核心的信息化基础设施建设，建设“中国扬州”门户网站和电子政务网络系统，构建城市GIS数据平台。加快港口物流园区建设，着力构造包括信息网络、海关监管、商检、税务等为主要内容的支撑系统，积极引进国内外知名物流企业落户扬州。举办地方特色产品、上海房车联展、服装面料、机床模具等展销会或博览会，形成扬州会展的品牌特色。推进社区服务产业化、品牌化、连锁化经营；鼓励各种经济成份发展社区和农村连锁服务业，改善城乡消费服务。县（市）区域经济是全市经济的重要组成部分，必须搞好区域生产力的合理布局，加快工业化、城镇化步伐，发展特色经济，形成区域经济板块，增强县（市）区级经济实力和政府的综合调控能力。

4、以完善功能、提升形象为着力点，争创城市环境新优势。

以创建“国家园林城市”、“中国最佳人居环境奖”和争创“国家生态示范城市”为目标，通过集中开发、集约开发、集合开发，突出重点、解决难点、做出亮点，进一步完善城市综合功能。精心实施市区“十大工程”。通过今年和今后一段时间的努力，建设好以下工程：一是古运河景观工程。高起点规划黄金坝至通扬桥段古运河景观，重点实施跃进桥—解放桥—洼字街一线开发，建设双瓮城遗址公园、南门遗址公园，整修吴道台古宅等。二是古城历史文化街区保护和利用工程。重点实施东圈门—东关街历史文化街区保护和教场地段整治工程。规划何园—小盘谷—二分明月楼一线名园古宅和南河下盐商文化街区整治工程等。三是西部新区建设工程。延伸文昌西路至西北绕城互通，建设博物馆、火车站和商贸、居住配套设施。四是规划建设扬子津科技教育园区。整合一批学校进园发展，引进一批院校和研发机构进区孵化开发。五是市政道路整治工程。重点实施运河北路、江都路、解放路改造；延伸汶河北路至漕河路，漕河路西延至友谊路，平山堂路西延至邗江路，邗江路北延至维扬路；改建贾七路，延伸文汇西路；改造大学路、扬子江北路；主城区内改造一批街巷。拓宽改造北门桥、凤凰桥，兴建三湾大桥、便益门大桥。城市外围重点实施扬天、扬菱、扬冶路改建延伸至北绕城公路。六是水环境整治工程。重点整治二道河、漕河、玉带河等河道，改造沿岸景观。七是“绿杨城郭、生态扬州”工程。实施润扬大桥北接线，西北绕城公路、南绕城公路、宁启铁路城区段，廖家沟，京杭大运河百里绿色长廊和扬子津城市生态森林、润扬大桥森林公园的“六线两块”绿化工程。八是旅游景区、景点及广场建设工程。规划启动瘦西湖景区东扩工程，建设维扬广场、友谊广场、大水湾广场、圣心广场、便益门广场和文昌广场二期等。九是街景亮化、庄台改造工程。着力提升文昌路和汶河路、江阳路部分路段的街景档次，对盐阜路、泰州路等中心地段实施美化亮化；改造“城中村”20万平方米。十是公用事业工程。继续实施污水处理、热电联供工程，启动天然气项目，加快自来水、煤气管网的改造和延伸。实施好江都、高邮、宝应、仪征污水处理厂等一批工程。加强城市长效管理。巩固“三创”成果，重点抓好占道经营、餐饮业污染、烟囱和广告牌、店牌等专项整治，加强垃圾和交通秩序管理，美化、净化市容。强化社区建设，建立扎实的城市管理基础。推动城市管理向城郊辐射，市区周边的汤汪、湾头、蒋王、汊河、西湖、平山、施桥等7个乡镇，年内创建成省级卫生镇，各县（市）要争取1-2个镇成为省级卫生镇。完善城市建设与管理体制。贯彻“一级规划、二级建设、三级管理、四级服务”的要求，强化城市总体规划和功能分区规划的修编、实施。理顺市、区之间建管关系，提高区级政府“发展经济、管理城市、服务百姓”的水平。完善城建投融资体制，加大经营城市力度，在坚持土地统一管理、经营，增加资源性收入的同时，盘活存量，实施一部分市政公用基础设施专营权有偿转让。城建控股公司按企业运作，扩大投融资能力。

5、以建设“文化扬州”为抓手，促进社会事业新发展。

文化是城市之根、城市之魂，是城市的品位和竞争力所在。扬州文化底蕴深厚，建设文化扬州是贯彻“三个代表”重要思想、全面建设小康社会的需要，也是建设“古代文化与现代文明交相辉映的名城”和文化大市的需要。

全力建设“文化扬州”，着力开展四个方面工作：一是充分保护扬州历史文化资源，延续历史脉络。按照“护其貌、美其颜、扬其韵、铸其魂”的思路，对扬州文化独特个性及其优秀文化资源进行分析、整理，高起点制定古城保护利用、文化建设发展和旅游开发互动项目规划与实施方案，延伸文脉，彰显个性。二是重点抓好历史文化街区保护和整治，做大做优文化旅游产业。根据市人大常委会审定的《扬州市老城区控制性详细规划大纲》，实施对5.09平方公里老城区的保护，按照“打造一批特色街区、整合一批古老街巷、建设一批遗址公园、开辟一批展览馆所、恢复一批历史遗迹、修缮一批知名园宅、弘扬一批老字店号”的要求，成片保护，连片成线，营造浓郁的文化氛围，使老城区成为一个开放式历史博览、文化展示之城，进一步提升“文化扬州”品牌的知名度、美誉度。三是保护和展示非物质形态文化，弘扬特色文化。整合资源，规划兴办一批表演展示场所，常年表演展示有扬州特色的工艺美术、扬剧、评话、木偶、歌舞、书画等，丰富市民和旅游者的生活。鼓励创作有浓郁地方特色和时代特色的精品力作。建立扬州文化研究所，加强对扬州文化的研究。四是发展文化产业，创新扬州文化。落实文化经济政策，建立多元投入和企业运作机制，推进文化产业化。加快广陵书社运转。大力开展文化展示、文化交流活动，活跃群众文化生活。通过“文化扬州"建设，使扬州文化成为有活力的文化，扬州旅游成为有文化含量的旅游，扬州古城成为充满生机的古城。加大资源整合和设施建设力度。完成第一人民医院与第四人民医院、妇幼保健院与红十字医院的资源重组。积极推进县乡妇保机构、卫生院与同级计生指导所（站）的联合。抓好扬州博物馆和雕版印刷博物馆建设；加快职大电大新校区和市广播电视网络数字化、苏北人民医院改造工程、市中心血站综合楼等建设。规划启动新区体育公园。加快事业单位改革步伐，对非经营性事业单位，推行内部机制的改革，增强发展活力；对经营性的事业单位，加快建立现代企业制度，推进企业化运作。提高社会事业发展水平。坚持走科技与经济结合的路子，积极推进产学研联合，建立健全促进科技创新的政策和投入保障体制；完善人才政策，引进高层次人才，发挥高新技术创业服务中心作用，鼓励海外学子及各类人才来我市创业。组织实施省级以上“火炬”、“星火”项目60项，其中国家级30项。继续巩固九年义务教育的成果，促进高中教育、职业技术教育等各类教育协调发展。推进教育改革和教育信息化建设，提高教育教学质量。加强农村卫生服务网络、卫生队伍、卫生管理体制和医疗保障制度等建设，农村合作医疗覆盖率提高到70%，自来水普及率达90%，卫生厕所普及率达60%。开展人口与计划生育优质服务，提高人口素质。扩大群众性体育的普及面，备战十六届省运会，承办好亚洲艺术体操锦标赛和全国足球甲B联赛江苏舜天队扬州主场比赛等。深入开展精神文明创建活动，提高市民素质。继续组织实施“现代市民教育工程”和“现代农民教育工程”，广泛开展公民道德教育，制定、完善市民守则和乡规民约，开展创建“文明社区”、“文明单位”、“文明家庭”、“文明市民”活动，全面提高人民群众的道德素养，培养科学、文明、健康的市民习惯，塑造温文尔雅、文明健康、朝气蓬勃的市民形象。努力打造“诚信扬州”。加强全民国防教育和国防动员工作，争创“全国双拥模范城”四连冠。

6、以让老百姓“安居乐业”为落脚点，解决民生问题取得新进展。

大力推进就业再就业工作。以“为下岗失业人员找到一份工作”为目标，大力改善创业环境，加大困难群体再就业工作力度。全年新增就业岗位4万个以上，新安置再就业人员2万名，重点解决“4045”下岗职工和“双下岗”、军嫂、劳模、城镇退伍军人等优先就业。支持企业通过主辅分离、辅业转制，分流安置企业富余人员。继续推行政府购买岗位、购买培训成果，为困难群体实行免费培训。鼓励发展具有比较优势的劳动密集型企业、中小企业和劳服企业，增加就业岗位。扩大劳务输出规模，提升对外劳务协作层次。鼓励下岗失业人员自谋职业、自主创业。以“两个确保”为目标，增强社会保障能力。进一步加大基本养老保险的扩面、续保、征缴、清欠等工作力度，收缴率95%以上。加强对非国有企业的参保扩面工作，参保率扩大到35%左右，全市扩大参保5万人以上。稳妥推进下岗保障向失业保险并轨，积极完善失业保险制度，提高社会保障支出占财政支出的比重。加大再就业资金筹集力度，切实保障下岗职工基本生活。认真做好“三条保障线”的衔接，及时将所有符合低保条件的城乡居民纳入低保范围，做到应保尽保。高度重视解决困难企业到龄职工办理退休和拖欠职工工资、费用等遗留问题。做好农村扶贫工作。继续开展帮扶经济薄弱村和人均收入低于1200元的困难户活动，突出抓好村级经济和农民脱贫致富项目，帮助困难家庭树立信心，掌握适用技术，增强致富能力，让他们尽快走出困境。市、县（市、区）、镇共同筹资，年内解决1000户特困农民的住房问题。

做好新一轮住房解困解危工作。继续贯彻落实住房保障制度，切实解决弱势群体的住房困难，全市完成22万平方米危房解危任务；市区建设6万平方米定销房，让1000户人均住房低于8平方米、月收入低于300元的特困户“居者有其屋”。

积极稳妥推进城镇医疗保险制度改革。全市城镇职工基本医疗保险覆盖面80%以上，进一步提高医疗保障水平。修订完善困难企业职工及其退休人员参加基本医疗保险制度的办法，继续对下岗职工、低保对象实施大病统筹，在全市所有非盈利性医疗机构设立“慈济窗口”，对特困职工、重点优抚对象、农村五保户和贫困家庭实行大病医疗救助，让困难群体病有所医。

继续为群众办实事。今年市区重点抓好以下几方面的实事：安置下岗失业人员1万人，新增就业岗位2万个，养老保险扩面3万人；改造危房11万平方米；实施洼子街、石狮小区改造；在主城区街巷凡是土路全部硬化、凡是无路灯的全部亮化，翻建街巷50条；建设一批市民广场（公园）；实施生态扬州绿化工程，城区新增绿地100万平方米；改造一批道路、河道；完善一批城市管理卫生设施；抓好放心菜、放心粮生产和供应。

大力推进政府工作创新

今年是贯彻落实党的十六大精神的第一年，也是新一届政府开展工作的第一年。我们要认真落实党的十六大精神，全面贯彻“三个代表”重要思想，努力发扬“勤政务实高效”的良好政风，不断开创政府工作新局面。

解放思想，创造加快发展的环境。党的十六大提出了新的发展目标，我们正处在一个重要的发展阶段。实践证明，每一次经济大发展，都伴随着思想的大解放，在解放思想中统一思想，在解放思想中加快发展。政府和各部门工作人员都要“自觉地把思想认识从那些不合时宜的观念、做法和体制的束缚中解放出来，从对马克思主义错误的和教条式的理解中解放出来”，坚决冲破一切妨碍发展的思想观念，坚决改变一切束缚发展的做法和规定，坚决革除一切影响发展的体制弊端，破除小胜即满、小富即安的小城意识，倡导不断进取、争创一流的创新精神；破除怕担风险、甘居中游的平庸思想，树立奋发有为、敢为人先的开拓精神；破除怕苦畏难、无所作为的散漫作风，树立知难而进、乐于奉献的拼搏精神；破除本位主义、各自为政的狭隘观念，树立顾全大局、同心协力的团队精神，营造有利于新突破、新发展的浓烈氛围。在放开搞活中促进发展，全市上下必须彻底放开手脚、放低门槛、放活体制、放宽政策、放活要素，在思想上想放，工作上敢放，实践中会放，以开明的态度、开放的心态、开阔的胸怀，开渠放水，“让一切劳动、知识、技术、管理和资本的活力竞相迸发，让一切创造社会财富的源泉充分涌流”，让各种生产要素、资源加速向扬州聚集，使我市成为投资创业的宝地。

与时俱进，建设学习型政府。政府及其工作人员要顺应时代前进的要求，坚持自觉学习、终身学习、带头学习。学习邓小平理论和江泽民“三个代表”重要思想，用十六大精神指导政府的各项工作。学习文化科技知识，学习市场经济和法律、现代科技、行政管理等知识，熟悉和掌握世贸组织规则，增强市场化、国际化、现代化的观念。通过学习，提高科学判断形势的能力，提高驾驭经济全局的能力，提高依法行政的能力，提高服务民众的能力，提高政府的行政效率，推动学习型社会的建设。

依法行政，建设法制型政府。自觉接受市人大及其常委会的监督，依法报告工作，认真执行和落实有关决议、决定。积极支持人民政协履行政治协商、民主监督、参政议政的职能。主动向各民主党派、工商联通报情况，听取意见和建议，发挥工会、共青团、妇联等人民团体的作用。认真办理人大代表建议和政协委员提案。继续推进依法治市，认真搞好“四五”普法教育，增强全民法治观念。完善行政执法责任制和错案追究责任制，抓好执法队伍建设，进一步规范行政执法行为。加强基层民主建设，继续大力推进政务、厂务、村务公开。贯彻打防结合、预防为主的方针，建立完善打防控一体化体系，加强基层基础工作，全面落实社会治安综合治理各项措施，依法严厉打击各种刑事犯罪和经济犯罪活动，严防境内外敌对势力、法轮功等邪教组织的渗透、破坏活动，创建最安全的社会发展环境。贯彻落实党的民族宗教政策，做好侨务、对台工作。重视做好信访工作。加强安全生产。各级政府努力把职能转移到引导、规范、监管、服务上来，规范行政行为，提高行政效率。进一步整顿和规范市场经济秩序，依法加强税收征管，规范行政事业性收费，优化投资软环境。

牢记“两个务必”，建设服务型政府。各级政府要身体力行“三个代表”重要思想，增强忧患意识，务必保持谦虚谨慎、不骄不躁的作风，务必保持艰苦奋斗的作风，做到深怀爱民之心，恪守为民之责，善谋富民之策，多办利民之事。进一步畅通与民众沟通、联系的渠道，倾听民声，了解民意，掌握民情，善待民众，纾解民困，凝聚民心。继续办好“寄语市长”热线栏目，完善市长与市民“绿色通道”；健全政府工作部门负责人与人大代表、市民代表见面制度，主动深入基层，体察社情民意，掌握第一手资料。继续推行首问负责制、服务承诺制，做到急事急办、特事特办、难事巧办。加快电子政务建设，推行政务公开，向社会公开政府规范性文件、行政措施和政务活动，保障群众的知情权、参与权和监督权。围绕政府重点工作和事关群众切身利益的重大事项，抓紧建立和完善听证制度。

坚持反腐倡廉，建设廉洁高效型政府。切实加强廉政教育，进一步抓好领导干部廉洁自律，规范政府工作人员特别是领导干部的从政行为；坚持从严治政，加大查处违纪违法案件的力度；继续大力纠正部门和行业不正之风，解决群众反映的突出问题；抓住关键部位和环节，加大从源头上预防和治理腐败的力度；认真落实廉政建设责任制，形成齐抓共管的局面。依法加强国有资产管理。推行政府采购制度、招投标制度和部门预算，认真落实“收支两条线”规定；大力做好增收节支工作，严肃财经纪律，切实精减会议和文件，减少应酬活动，坚决制止铺张浪费，确保集中精力抓大事，集中财力搞建设。

各位代表，新的目标鼓舞人心，新的事业任重道远，新的形势催人奋进。让我们在党的十六大精神指引下，高举邓小平理论伟大旗帜，全面贯彻“三个代表”重要思想，解放思想，抢抓机遇，与时俱进，开拓创新，同心同德，埋头苦干，为开创扬州改革发展新局面，创造更加美好的未来而努力奋斗！
